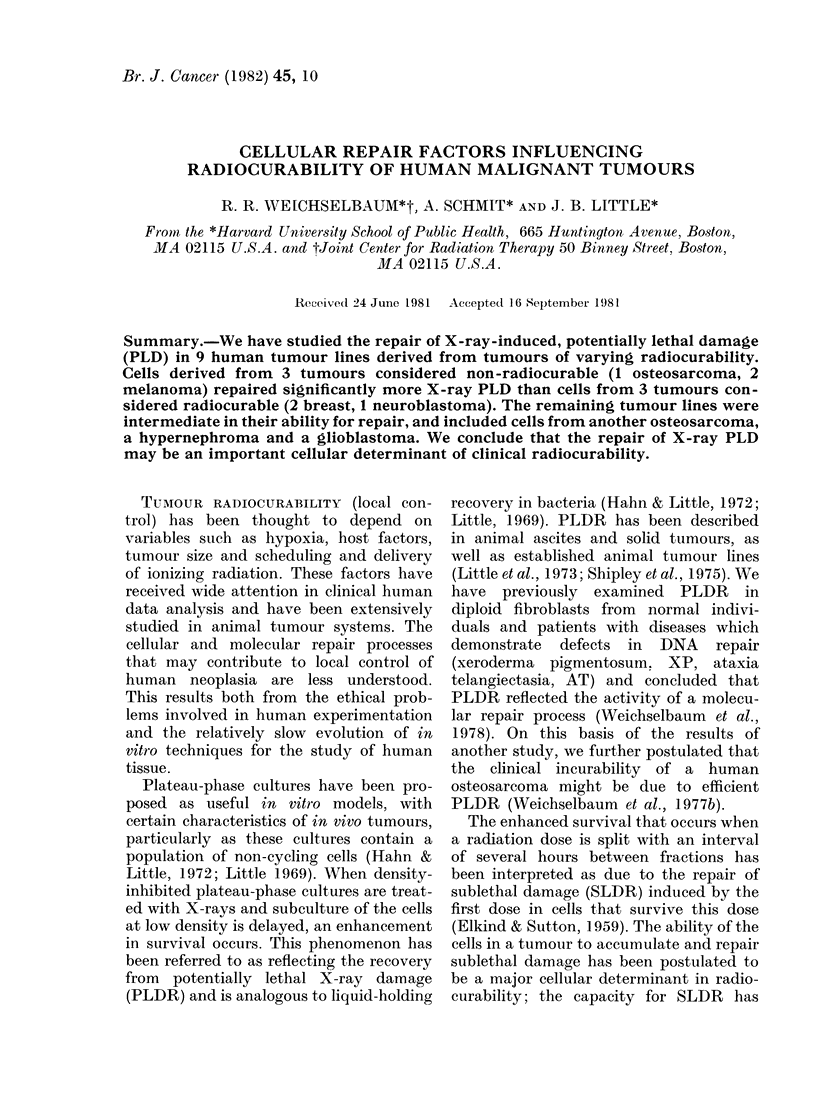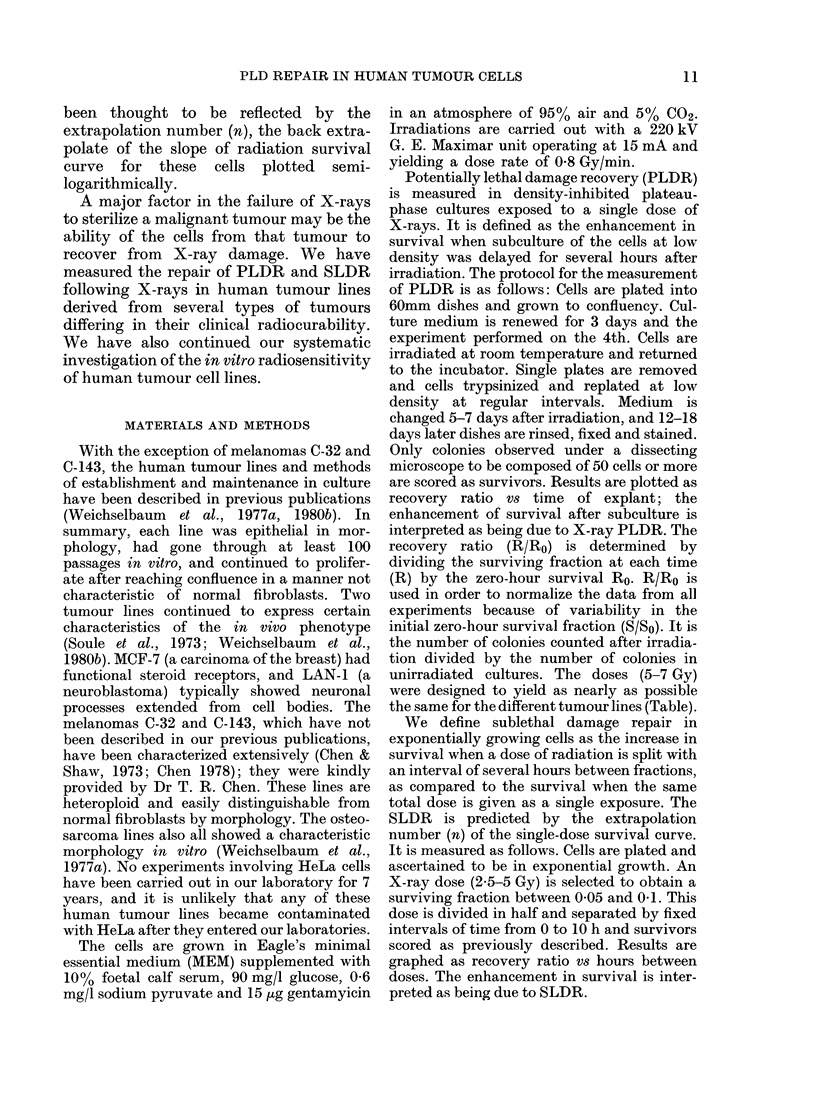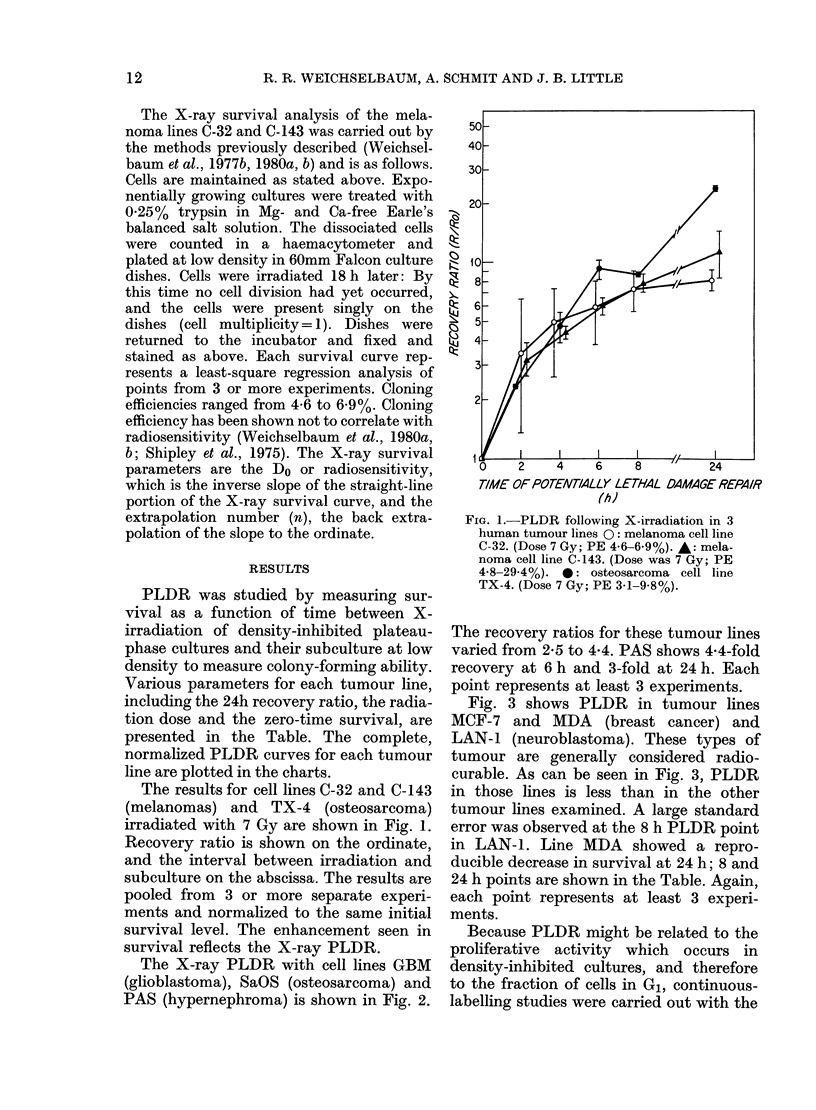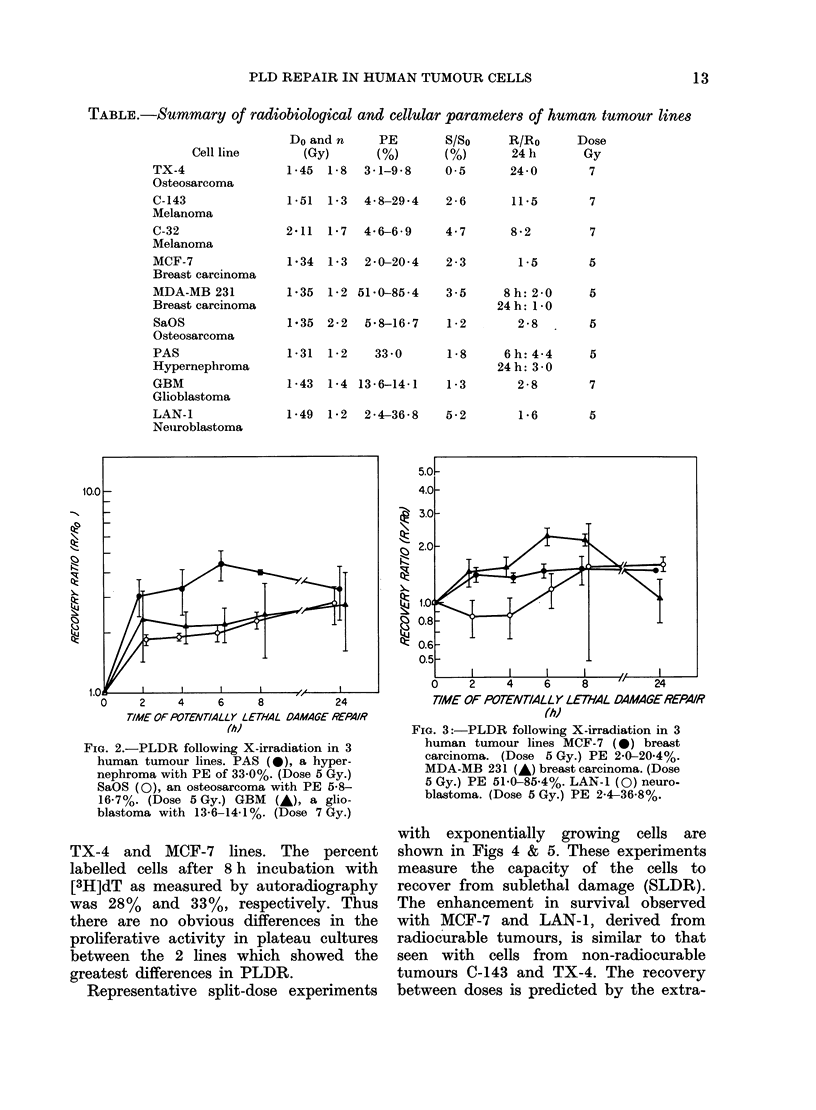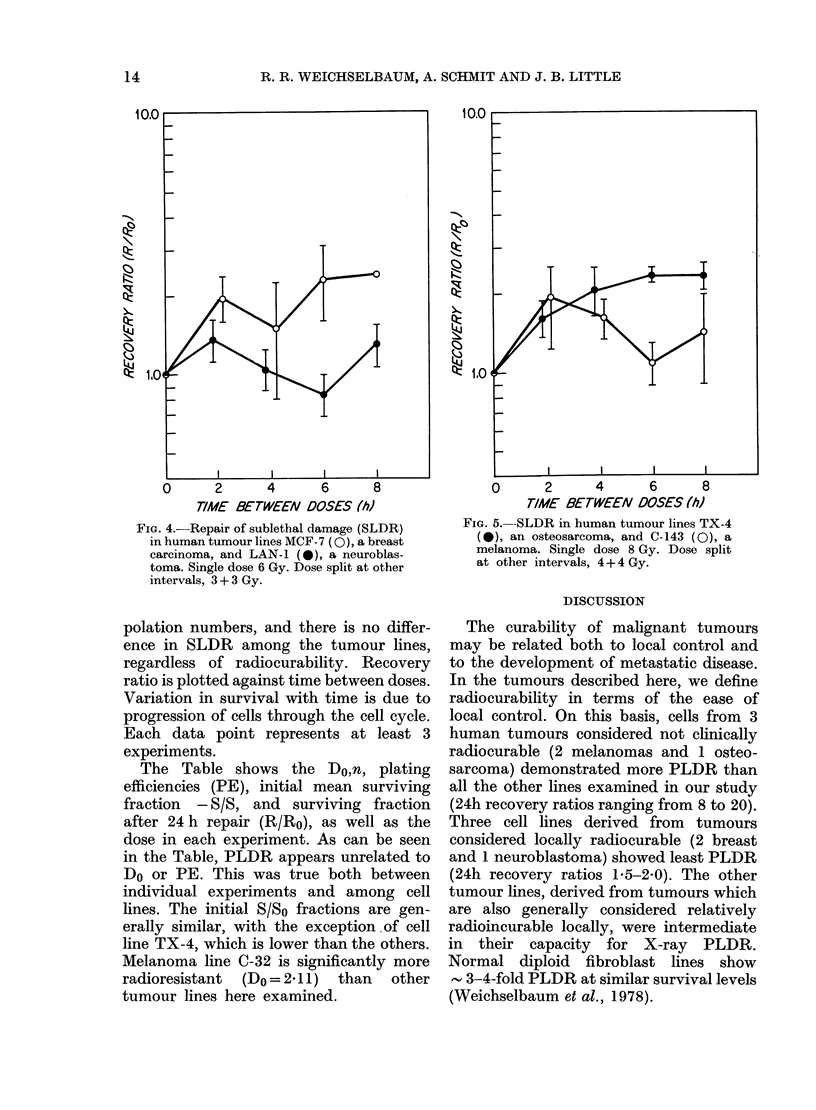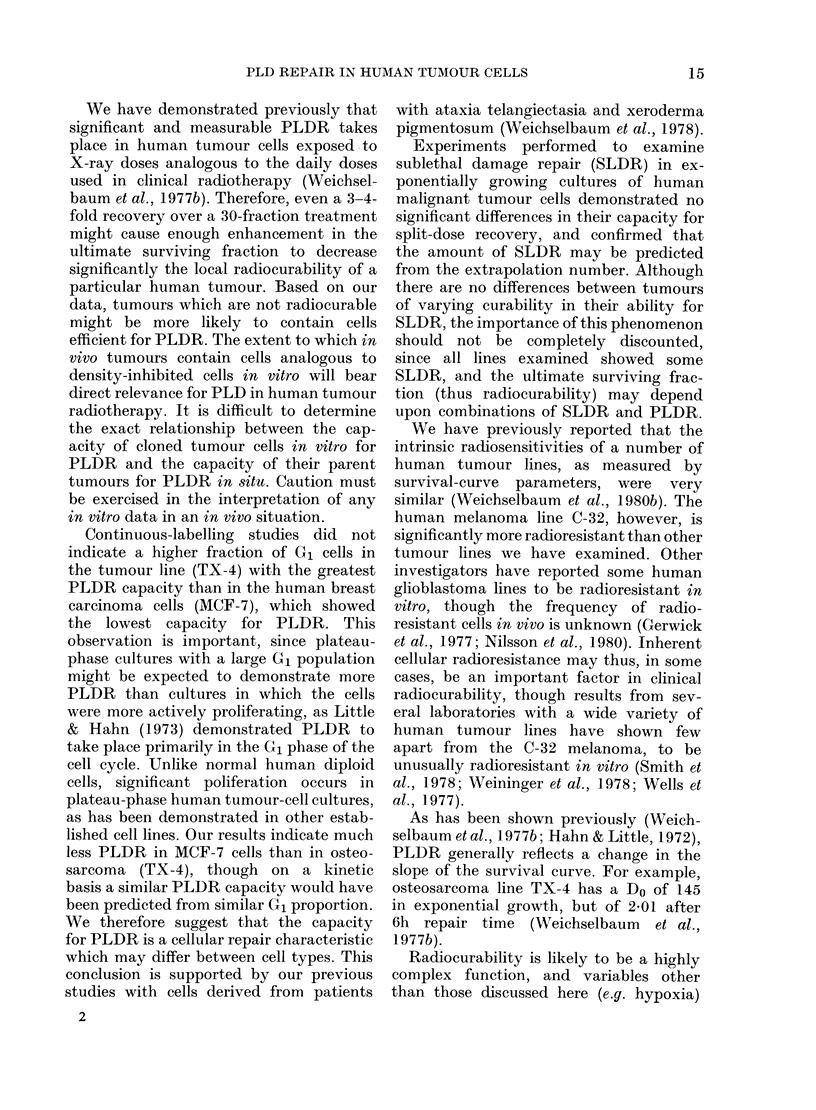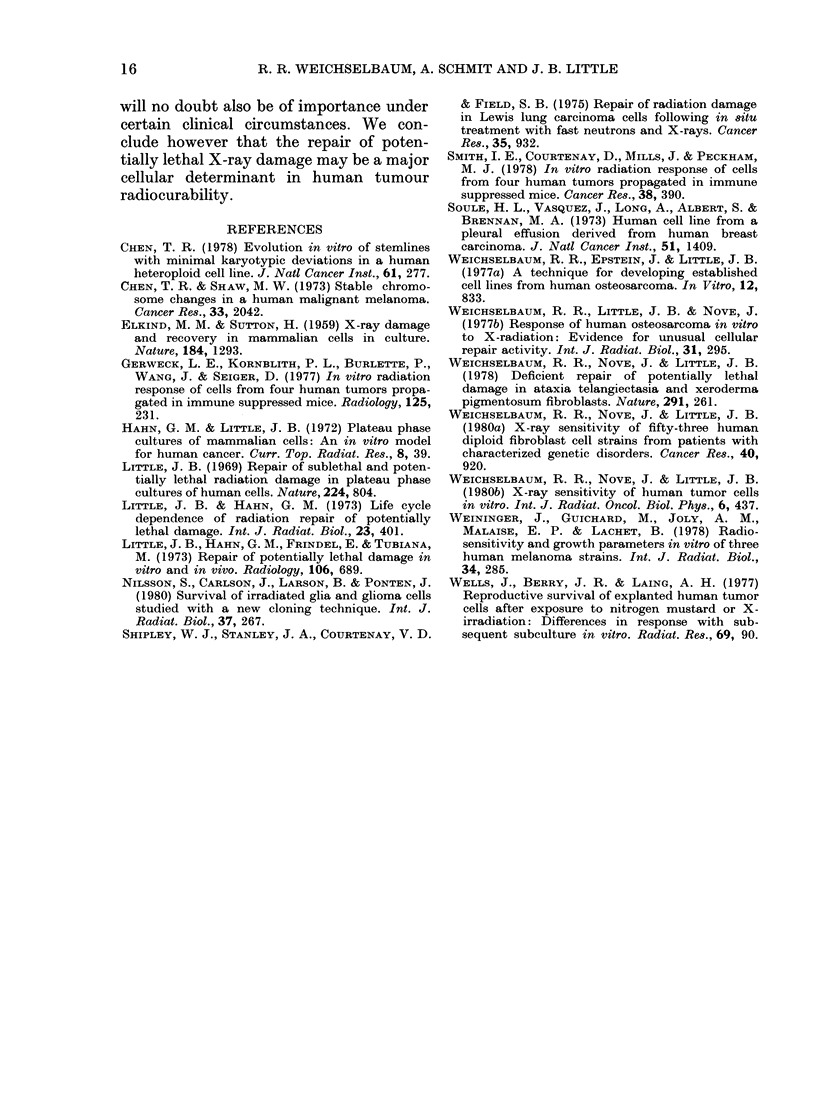# Cellular repair factors influencing radiocurability of human malignant tumours.

**DOI:** 10.1038/bjc.1982.2

**Published:** 1982-01

**Authors:** R. R. Weichselbaum, A. Schmit, J. B. Little

## Abstract

We have studied the repair of X-ray-induced, potentially lethal damage (PLD) in 9 human tumour lines derived from tumours of varying radiocurability. Cells derived from 3 tumours considered non-radiocurable (1 osteosarcoma, 2 melanoma) repaired significantly more X-ray PLD than cells from 3 tumours considered radiocurable (2 breast, 1 neuroblastoma). The remaining tumour lines were intermediate in their ability for repair, and included cells from another osteosarcoma, a hypernephroma and a glioblastoma. We conclude that the repair of X-ray PLD may be an important cellular determinant of clinical radiocurability.


*Br. J. Cancer* (1982) **45**, 10

### CELLULAR REPAIR FACTORS INFLUENCING RADIOCURABILITY OF HUMAN MALIGNANT TUMOURS

R. R. WEICHSELBAUM*†, A. SCHMIT* AND J. B. LITTLE*

*From the *Harvard University School of Public Health,* 665 *Huntington Avenue, Boston, MA* 02115 *U.S.A. and †Joint Center for Radiation Therapy* 50 *Binney Street, Boston, MA* 02115 *U.S.A.*

Received 24 June 1981 Accepted 16 September 1981

**Summary.—We have studied the repair of X-ray-induced, potentially lethal damage (PLD) in 9 human tumour lines derived from tumours of varying radiocurability. Cells derived from 3 tumours considered non-radiocurable (1 osteosarcoma, 2 melanoma) repaired significantly more X-ray PLD than cells from 3 tumours considered radiocurable (2 breast, 1 neuroblastoma). The remaining tumour lines were intermediate in their ability for repair, and included cells from another osteosarcoma, a hypernephroma and a glioblastoma. We conclude that the repair of X-ray PLD may be an important cellular determinant of clinical radiocurability.**

TUMOUR RADIOCURABILITY (local control) has been thought to depend on variables such as hypoxia, host factors, tumour size and scheduling and delivery of ionizing radiation. These factors have received wide attention in clinical human data analysis and have been extensively studied in animal tumour systems. The cellular and molecular repair processes that may contribute to local control of human neoplasia are less understood. This results both from the ethical problems involved in human experimentation and the relatively slow evolution of *in vitro* techniques for the study of human tissue.

Plateau-phase cultures have been proposed as useful *in vitro* models, with certain characteristics of *in vivo* tumours, particularly as these cultures contain a population of non-cycling cells (Hahn & Little, 1972; Little 1969). When density-inhibited plateau-phase cultures are treated with X-rays and subculture of the cells at low density is delayed, an enhancement in survival occurs. This phenomenon has been referred to as reflecting the recovery from potentially lethal X-ray damage (PLDR) and is analogous to liquid-holding recovery in bacteria (Hahn & Little, 1972; Little, 1969). PLDR has been described in animal ascites and solid tumours, as well as established animal tumour lines (Little *et al.*, 1973; Shipley *et al.*, 1975). We have previously examined PLDR in diploid fibroblasts from normal individuals and patients with diseases which demonstrate defects in DNA repair (xeroderma pigmentosum, XP, ataxia telangiectasia, AT) and concluded that PLDR reflected the activity of a molecular repair process (Weichselbaum *et al.*, 1978). On this basis of the results of another study, we further postulated that the clinical incurability of a human osteosarcoma might be due to efficient PLDR (Weichselbaum *et al.*, 1977*b*).

The enhanced survival that occurs when a radiation dose is split with an interval of several hours between fractions has been interpreted as due to the repair of sublethal damage (SLDR) induced by the first dose in cells that survive this dose (Elkind & Sutton, 1959). The ability of the cells in a tumour to accumulate and repair sublethal damage has been postulated to be a major cellular determinant in radiocurability; the capacity for SLDR has been thought to be reflected by the extrapolation number ($n$), the back extrapolate of the slope of radiation survival curve for these cells plotted semilogarithmically.

A major factor in the failure of X-rays to sterilize a malignant tumour may be the ability of the cells from that tumour to recover from X-ray damage. We have measured the repair of PLDR and SLDR following X-rays in human tumour lines derived from several types of tumours differing in their clinical radiocurability. We have also continued our systematic investigation of the *in vitro* radiosensitivity of human tumour cell lines.

### MATERIALS AND METHODS

With the exception of melanomas C-32 and C-143, the human tumour lines and methods of establishment and maintenance in culture have been described in previous publications (Weichselbaum *et al.*, 1977*a*, 1980*b*). In summary, each line was epithelial in morphology, had gone through at least 100 passages *in vitro*, and continued to proliferate after reaching confluence in a manner not characteristic of normal fibroblasts. Two tumour lines continued to express certain characteristics of the *in vivo* phenotype (Soule *et al.*, 1973; Weichselbaum *et al.*, 1980*b*). MCF-7 (a carcinoma of the breast) had functional steroid receptors, and LAN-1 (a neuroblastoma) typically showed neuronal processes extended from cell bodies. The melanomas C-32 and C-143, which have not been described in our previous publications, have been characterized extensively (Chen & Shaw, 1973; Chen 1978); they were kindly provided by Dr T. R. Chen. These lines are heteroploid and easily distinguishable from normal fibroblasts by morphology. The osteosarcoma lines also all showed a characteristic morphology *in vitro* (Weichselbaum *et al.*, 1977*a*). No experiments involving HeLa cells have been carried out in our laboratory for 7 years, and it is unlikely that any of these human tumour lines became contaminated with HeLa after they entered our laboratories.

The cells are grown in Eagle's minimal essential medium (MEM) supplemented with 10% foetal calf serum, 90 mg/l glucose, 0·6 mg/l sodium pyruvate and 15 $\mu$g gentamyicin in an atmosphere of 95% air and 5% $CO_2$. Irradiations are carried out with a 220 kV G. E. Maximar unit operating at 15 mA and yielding a dose rate of 0·8 Gy/min.

Potentially lethal damage recovery (PLDR) is measured in density-inhibited plateau-phase cultures exposed to a single dose of X-rays. It is defined as the enhancement in survival when subculture of the cells at low density was delayed for several hours after irradiation. The protocol for the measurement of PLDR is as follows: Cells are plated into 60mm dishes and grown to confluency. Culture medium is renewed for 3 days and the experiment performed on the 4th. Cells are irradiated at room temperature and returned to the incubator. Single plates are removed and cells trypsinized and replated at low density at regular intervals. Medium is changed 5–7 days after irradiation, and 12–18 days later dishes are rinsed, fixed and stained. Only colonies observed under a dissecting microscope to be composed of 50 cells or more are scored as survivors. Results are plotted as recovery ratio *vs* time of explant; the enhancement of survival after subculture is interpreted as being due to X-ray PLDR. The recovery ratio ($R/R_0$) is determined by dividing the surviving fraction at each time (R) by the zero-hour survival $R_0$. $R/R_0$ is used in order to normalize the data from all experiments because of variability in the initial zero-hour survival fraction ($S/S_0$). It is the number of colonies counted after irradiation divided by the number of colonies in unirradiated cultures. The doses (5–7 Gy) were designed to yield as nearly as possible the same for the different tumour lines (Table).

We define sublethal damage repair in exponentially growing cells as the increase in survival when a dose of radiation is split with an interval of several hours between fractions, as compared to the survival when the same total dose is given as a single exposure. The SLDR is predicted by the extrapolation number ($n$) of the single-dose survival curve. It is measured as follows. Cells are plated and ascertained to be in exponential growth. An X-ray dose (2·5–5 Gy) is selected to obtain a surviving fraction between 0·05 and 0·1. This dose is divided in half and separated by fixed intervals of time from 0 to 10 h and survivors scored as previously described. Results are graphed as recovery ratio *vs* hours between doses. The enhancement in survival is interpreted as being due to SLDR.

The X-ray survival analysis of the melanoma lines C-32 and C-143 was carried out by the methods previously described (Weichselbaum *et al.*, 1977*b*, 1980*a*, *b*) and is as follows. Cells are maintained as stated above. Exponentially growing cultures were treated with 0·25% trypsin in Mg- and Ca-free Earle's balanced salt solution. The dissociated cells were counted in a haemacytometer and plated at low density in 60mm Falcon culture dishes. Cells were irradiated 18 h later: By this time no cell division had yet occurred, and the cells were present singly on the dishes (cell multiplicity = 1). Dishes were returned to the incubator and fixed and stained as above. Each survival curve represents a least-square regression analysis of points from 3 or more experiments. Cloning efficiencies ranged from 4·6 to 6·9%. Cloning efficiency has been shown not to correlate with radiosensitivity (Weichselbaum *et al.*, 1980*a*, *b*; Shipley *et al.*, 1975). The X-ray survival parameters are the $D_0$ or radiosensitivity, which is the inverse slope of the straight-line portion of the X-ray survival curve, and the extrapolation number (*n*), the back extrapolation of the slope to the ordinate.

FIG. 1.—PLDR following X-irradiation in 3 human tumour lines ○: melanoma cell line C-32. (Dose 7 Gy; PE 4·6–6·9%). ▲: melanoma cell line C-143. (Dose was 7 Gy; PE 4·8–29·4%). ●: osteosarcoma cell line TX-4. (Dose 7 Gy; PE 3·1–9·8%).

### RESULTS

PLDR was studied by measuring survival as a function of time between X-irradiation of density-inhibited plateau-phase cultures and their subculture at low density to measure colony-forming ability. Various parameters for each tumour line, including the 24h recovery ratio, the radiation dose and the zero-time survival, are presented in the Table. The complete, normalized PLDR curves for each tumour line are plotted in the charts.

The results for cell lines C-32 and C-143 (melanomas) and TX-4 (osteosarcoma) irradiated with 7 Gy are shown in Fig. 1. Recovery ratio is shown on the ordinate, and the interval between irradiation and subculture on the abscissa. The results are pooled from 3 or more separate experiments and normalized to the same initial survival level. The enhancement seen in survival reflects the X-ray PLDR.

The X-ray PLDR with cell lines GBM (glioblastoma), SaOS (osteosarcoma) and PAS (hypernephroma) is shown in Fig. 2. The recovery ratios for these tumour lines varied from 2·5 to 4·4. PAS shows 4·4-fold recovery at 6 h and 3-fold at 24 h. Each point represents at least 3 experiments.

Fig. 3 shows PLDR in tumour lines MCF-7 and MDA (breast cancer) and LAN-1 (neuroblastoma). These types of tumour are generally considered radiocurable. As can be seen in Fig. 3, PLDR in those lines is less than in the other tumour lines examined. A large standard error was observed at the 8 h PLDR point in LAN-1. Line MDA showed a reproducible decrease in survival at 24 h; 8 and 24 h points are shown in the Table. Again, each point represents at least 3 experiments.

Because PLDR might be related to the proliferative activity which occurs in density-inhibited cultures, and therefore to the fraction of cells in $G_1$, continuous-labelling studies were carried out with the

TABLE.—*Summary of radiobiological and cellular parameters of human tumour lines*

| Cell line | $D_0$ and $n$ (Gy) | | PE (%) | $S/S_0$ (%) | $R/R_0$ 24 h | Dose Gy |
|---|---|---|---|---|---|---|
| TX-4 Osteosarcoma | 1·45 | 1·8 | 3·1–9·8 | 0·5 | 24·0 | 7 |
| C-143 Melanoma | 1·51 | 1·3 | 4·8–29·4 | 2·6 | 11·5 | 7 |
| C-32 Melanoma | 2·11 | 1·7 | 4·6–6·9 | 4·7 | 8·2 | 7 |
| MCF-7 Breast carcinoma | 1·34 | 1·3 | 2·0–20·4 | 2·3 | 1·5 | 5 |
| MDA-MB 231 Breast carcinoma | 1·35 | 1·2 | 51·0–85·4 | 3·5 | 8 h: 2·0<br>24 h: 1·0 | 5 |
| SaOS Osteosarcoma | 1·35 | 2·2 | 5·8–16·7 | 1·2 | 2·8 | 5 |
| PAS Hypernephroma | 1·31 | 1·2 | 33·0 | 1·8 | 6 h: 4·4<br>24 h: 3·0 | 5 |
| GBM Glioblastoma | 1·43 | 1·4 | 13·6–14·1 | 1·3 | 2·8 | 7 |
| LAN-1 Neuroblastoma | 1·49 | 1·2 | 2·4–36·8 | 5·2 | 1·6 | 5 |

FIG. 2.—PLDR following X-irradiation in 3 human tumour lines. PAS (●), a hypernephroma with PE of 33·0%. (Dose 5 Gy.) SaOS (○), an osteosarcoma with PE 5·8–16·7%. (Dose 5 Gy.) GBM (▲), a glioblastoma with 13·6–14·1%. (Dose 7 Gy.)

FIG. 3:—PLDR following X-irradiation in 3 human tumour lines MCF-7 (●) breast carcinoma. (Dose 5 Gy.) PE 2·0–20·4%. MDA-MB 231 (▲) breast carcinoma. (Dose 5 Gy.) PE 51·0–85·4%. LAN-1 (○) neuroblastoma. (Dose 5 Gy.) PE 2·4–36·8%.

TX-4 and MCF-7 lines. The percent labelled cells after 8 h incubation with [$^3$H]dT as measured by autoradiography was 28% and 33%, respectively. Thus there are no obvious differences in the proliferative activity in plateau cultures between the 2 lines which showed the greatest differences in PLDR.

Representative split-dose experiments with exponentially growing cells are shown in Figs 4 & 5. These experiments measure the capacity of the cells to recover from sublethal damage (SLDR). The enhancement in survival observed with MCF-7 and LAN-1, derived from radiocurable tumours, is similar to that seen with cells from non-radiocurable tumours C-143 and TX-4. The recovery between doses is predicted by the extra-

FIG. 4.—Repair of sublethal damage (SLDR) in human tumour lines MCF-7 (○), a breast carcinoma, and LAN-1 (●), a neuroblastoma. Single dose 6 Gy. Dose split at other intervals, 3+3 Gy.

FIG. 5.—SLDR in human tumour lines TX-4 (●), an osteosarcoma, and C-143 (○), a melanoma. Single dose 8 Gy. Dose split at other intervals, 4+4 Gy.

polation numbers, and there is no difference in SLDR among the tumour lines, regardless of radiocurability. Recovery ratio is plotted against time between doses. Variation in survival with time is due to progression of cells through the cell cycle. Each data point represents at least 3 experiments.

The Table shows the $D_0$,*n*, plating efficiencies (PE), initial mean surviving fraction $-S/S$, and surviving fraction after 24 h repair ($R/R_0$), as well as the dose in each experiment. As can be seen in the Table, PLDR appears unrelated to $D_0$ or PE. This was true both between individual experiments and among cell lines. The initial $S/S_0$ fractions are generally similar, with the exception of cell line TX-4, which is lower than the others. Melanoma line C-32 is significantly more radioresistant ($D_0=2{\cdot}11$) than other tumour lines here examined.

### DISCUSSION

The curability of malignant tumours may be related both to local control and to the development of metastatic disease. In the tumours described here, we define radiocurability in terms of the ease of local control. On this basis, cells from 3 human tumours considered not clinically radiocurable (2 melanomas and 1 osteosarcoma) demonstrated more PLDR than all the other lines examined in our study (24h recovery ratios ranging from 8 to 20). Three cell lines derived from tumours considered locally radiocurable (2 breast and 1 neuroblastoma) showed least PLDR (24h recovery ratios 1·5–2·0). The other tumour lines, derived from tumours which are also generally considered relatively radioincurable locally, were intermediate in their capacity for X-ray PLDR. Normal diploid fibroblast lines show ~ 3–4-fold PLDR at similar survival levels (Weichselbaum *et al.*, 1978).

We have demonstrated previously that significant and measurable PLDR takes place in human tumour cells exposed to X-ray doses analogous to the daily doses used in clinical radiotherapy (Weichselbaum *et al.*, 1977*b*). Therefore, even a 3–4-fold recovery over a 30-fraction treatment might cause enough enhancement in the ultimate surviving fraction to decrease significantly the local radiocurability of a particular human tumour. Based on our data, tumours which are not radiocurable might be more likely to contain cells efficient for PLDR. The extent to which *in vivo* tumours contain cells analogous to density-inhibited cells *in vitro* will bear direct relevance for PLD in human tumour radiotherapy. It is difficult to determine the exact relationship between the capacity of cloned tumour cells *in vitro* for PLDR and the capacity of their parent tumours for PLDR *in situ*. Caution must be exercised in the interpretation of any *in vitro* data in an *in vivo* situation.

Continuous-labelling studies did not indicate a higher fraction of $G_1$ cells in the tumour line (TX-4) with the greatest PLDR capacity than in the human breast carcinoma cells (MCF-7), which showed the lowest capacity for PLDR. This observation is important, since plateau-phase cultures with a large $G_1$ population might be expected to demonstrate more PLDR than cultures in which the cells were more actively proliferating, as Little & Hahn (1973) demonstrated PLDR to take place primarily in the $G_1$ phase of the cell cycle. Unlike normal human diploid cells, significant poliferation occurs in plateau-phase human tumour-cell cultures, as has been demonstrated in other established cell lines. Our results indicate much less PLDR in MCF-7 cells than in osteosarcoma (TX-4), though on a kinetic basis a similar PLDR capacity would have been predicted from similar $G_1$ proportion. We therefore suggest that the capacity for PLDR is a cellular repair characteristic which may differ between cell types. This conclusion is supported by our previous studies with cells derived from patients with ataxia telangiectasia and xeroderma pigmentosum (Weichselbaum *et al.*, 1978).

Experiments performed to examine sublethal damage repair (SLDR) in exponentially growing cultures of human malignant tumour cells demonstrated no significant differences in their capacity for split-dose recovery, and confirmed that the amount of SLDR may be predicted from the extrapolation number. Although there are no differences between tumours of varying curability in their ability for SLDR, the importance of this phenomenon should not be completely discounted, since all lines examined showed some SLDR, and the ultimate surviving fraction (thus radiocurability) may depend upon combinations of SLDR and PLDR.

We have previously reported that the intrinsic radiosensitivities of a number of human tumour lines, as measured by survival-curve parameters, were very similar (Weichselbaum *et al.*, 1980*b*). The human melanoma line C-32, however, is significantly more radioresistant than other tumour lines we have examined. Other investigators have reported some human glioblastoma lines to be radioresistant *in vitro*, though the frequency of radioresistant cells *in vivo* is unknown (Gerwick *et al.*, 1977; Nilsson *et al.*, 1980). Inherent cellular radioresistance may thus, in some cases, be an important factor in clinical radiocurability, though results from several laboratories with a wide variety of human tumour lines have shown few apart from the C-32 melanoma, to be unusually radioresistant *in vitro* (Smith *et al.*, 1978; Weininger *et al.*, 1978; Wells *et al.*, 1977).

As has been shown previously (Weichselbaum *et al.*, 1977*b*; Hahn & Little, 1972), PLDR generally reflects a change in the slope of the survival curve. For example, osteosarcoma line TX-4 has a $D_0$ of 145 in exponential growth, but of 2·01 after 6h repair time (Weichselbaum *et al.*, 1977*b*).

Radiocurability is likely to be a highly complex function, and variables other than those discussed here (*e.g.* hypoxia)

will no doubt also be of importance under certain clinical circumstances. We conclude however that the repair of potentially lethal X-ray damage may be a major cellular determinant in human tumour radiocurability.

### REFERENCES

CHEN, T. R. (1978) Evolution *in vitro* of stemlines with minimal karyotypic deviations in a human heteroploid cell line. *J. Natl Cancer Inst.*, **61**, 277.

CHEN, T. R. & SHAW, M. W. (1973) Stable chromosome changes in a human malignant melanoma. *Cancer Res.*, **33**, 2042.

ELKIND, M. M. & SUTTON, H. (1959) X-ray damage and recovery in mammalian cells in culture. *Nature*, **184**, 1293.

GERWECK, L. E., KORNBLITH, P. L., BURLETTE, P., WANG, J. & SEIGER, D. (1977) *In vitro* radiation response of cells from four human tumors propagated in immune suppressed mice. *Radiology*, **125**, 231.

HAHN, G. M. & LITTLE, J. B. (1972) Plateau phase cultures of mammalian cells: An *in vitro* model for human cancer. *Curr. Top. Radiat. Res.*, **8**, 39.

LITTLE, J. B. (1969) Repair of sublethal and potentially lethal radiation damage in plateau phase cultures of human cells. *Nature*, **224**, 804.

LITTLE, J. B. & HAHN, G. M. (1973) Life cycle dependence of radiation repair of potentially lethal damage. *Int. J. Radiat. Biol.*, **23**, 401.

LITTLE, J. B., HAHN, G. M., FRINDEL, E. & TUBIANA, M. (1973) Repair of potentially lethal damage *in vitro* and *in vivo*. *Radiology*, **106**, 689.

NILSSON, S., CARLSON, J., LARSON, B. & PONTEN, J. (1980) Survival of irradiated glia and glioma cells studied with a new cloning technique. *Int. J. Radiat. Biol.*, **37**, 267.

SHIPLEY, W. J., STANLEY, J. A., COURTENAY, V. D. & FIELD, S. B. (1975) Repair of radiation damage in Lewis lung carcinoma cells following *in situ* treatment with fast neutrons and X-rays. *Cancer Res.*, **35**, 932.

SMITH, I. E., COURTENAY, D., MILLS, J. & PECKHAM, M. J. (1978) *In vitro* radiation response of cells from four human tumors propagated in immune suppressed mice. *Cancer Res.*, **38**, 390.

SOULE, H. L., VASQUEZ, J., LONG, A., ALBERT, S. & BRENNAN, M. A. (1973) Human cell line from a pleural effusion derived from human breast carcinoma. *J. Natl Cancer Inst.*, **51**, 1409.

WEICHSELBAUM, R. R., EPSTEIN, J. & LITTLE, J. B. (1977*a*) A technique for developing established cell lines from human osteosarcoma. *In Vitro*, **12**, 833.

WEICHSELBAUM, R. R., LITTLE, J. B. & NOVE, J. (1977*b*) Response of human osteosarcoma *in vitro* to X-radiation: Evidence for unusual cellular repair activity. *Int. J. Radiat. Biol.*, **31**, 295.

WEICHSELBAUM, R. R., NOVE, J. & LITTLE, J. B. (1978) Deficient repair of potentially lethal damage in ataxia telangiectasia and xeroderma pigmentosum fibroblasts. *Nature*, **291**, 261.

WEICHSELBAUM, R. R., NOVE, J. & LITTLE, J. B. (1980*a*) X-ray sensitivity of fifty-three human diploid fibroblast cell strains from patients with characterized genetic disorders. *Cancer Res.*, **40**, 920.

WEICHSELBAUM, R. R., NOVE, J. & LITTLE, J. B. (1980*b*) X-ray sensitivity of human tumor cells *in vitro*. *Int. J. Radiat. Oncol. Biol. Phys.*, **6**, 437.

WEININGER, J., GUICHARD, M., JOLY, A. M., MALAISE, E. P. & LACHET, B. (1978) Radiosensitivity and growth parameters *in vitro* of three human melanoma strains. *Int. J. Radiat. Biol.*, **34**, 285.

WELLS, J., BERRY, J. R. & LAING, A. H. (1977) Reproductive survival of explanted human tumor cells after exposure to nitrogen mustard or X-irradiation: Differences in response with subsequent subculture *in vitro*. *Radiat. Res.*, **69**, 90.